# Fibrogenesis in chronic murine colitis is independent of innate lymphoid cells

**DOI:** 10.1002/iid3.321

**Published:** 2020-06-21

**Authors:** Brecht Creyns, Jonathan Cremer, Gert De Hertogh, Louis Boon, Marc Ferrante, Séverine Vermeire, Gert Van Assche, Jan L. Ceuppens, Christine Breynaert

**Affiliations:** ^1^ Department of Microbiology, Immunology and Transplantation, Allergy and Clinical Immunology Research Group KU Leuven Leuven Belgium; ^2^ Department of Chronic Diseases, Metabolism and Ageing Translational Research Center for Gastrointestinal Disorders (TARGID), KU Leuven Leuven Belgium; ^3^ Department of Imaging and Pathology, Translational Cell and Tissue Research KU Leuven Leuven Belgium; ^4^ Bioceros BV Utrecht The Netherlands; ^5^ Department of Gastroenterology and Hepatology University Hospitals Leuven, KU Leuven Leuven Belgium; ^6^ Department of General Internal Medicine University Hospitals Leuven, KU Leuven Leuven Belgium

**Keywords:** Crohn's disease, innate immunity, innate lymphoid cell, intestinal fibrosis

## Abstract

**Introduction:**

Insight in the pathogenesis of intestinal fibrosis is an unmet medical need in inflammatory bowel diseases. Studies in murine models and human organ fibrosis point to a potential role of innate lymphoid cells (ILC) in chronic intestinal inflammation and fibrosis.

**Materials and Methods:**

Dextran sodium sulfate (DSS) in drinking water was used to induce chronic colitis and remodeling in C57Bl/6 wild type (WT), RAG‐deficient, RAG^−/−^ common γ chain deficient and anti‐CD90.2 monoclonal antibody treated RAG^−/−^ mice. Inflammation was scored by macroscopic and histological examination and fibrosis was evaluated by hydroxyproline quantification and histology.

**Results:**

In RAG^−/−^ mice (which have a normal ILC population but no adaptive immunity), chronic intestinal inflammation and fibrosis developed similarly as in WT mice, with a relative increase in ILC2 during repeated DSS exposure. Chronic colitis could also be induced in the absence of ILC (RAG^−/−^γc^−/−^ or anti‐CD90.2 treated RAG^−/−^ mice) with no attenuation of fibrosis. Importantly, clinical recovery based on weight gain after stopping DSS exposure was impaired in ILC‐deficient or ILC‐depleted mice.

**Conclusion:**

These data argue against a profibrotic effect of ILC in chronic colitis, but rather suggest that ILC have a protective and recovery‐enhancing effect after repeated intestinal injury.

## Graphical abstract

Identification of innate lymphoid cells (ILC) as mucosal, resident, cytokine‐producing cells, and players in liver and lung fibrosis, led us to the study of these cells in our murine model of chronic intestinal inflammation and fibrosis. In both wild type (WT) and RAG1^−/−^ mice we observed an enrichment of interleukin (IL)‐13 producing ILC2 with increasing numbers of dextran sodium sulfate (DSS) administration. However, upon antibody‐mediated depletion of all ILC, no attenuation of fibrosis could be observed. These results were validated in ILC deficient mice. However, in these mice we found that in absence of ILC, recovery after withdrawal of DSS is impaired and associated with a higher mortality.

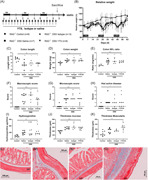

## 1 INTRODUCTION

Chronic inflammatory bowel diseases (IBD), comprising Crohn's disease (CD) and ulcerative colitis (UC), are inflammatory intestinal diseases with a relapsing‐remitting disease course. Throughout their disease course, one‐third of CD patients and 5% of UC patients will develop a fibrostenotic phenotype (strictures and bowel obstructions) due to excessive extracellular matrix deposition and muscularis propria hyperplasia in the bowel wall.^1^, ^2^ Up to 80% of CD and 20% of UC patients undergo surgery during the disease course, with stricture formation being the most common indication for major intestinal surgery in CD.^3^, ^4^ Notably, recurrence of inflammation and fibrosis after surgical resection is common. Although fibrosis is most prevalent in CD, a minority of patients with extensive UC of long duration have shortening of the colon with formation of a lead‐pipe colon as a result of excessive remodeling.^5^ The mechanisms by which fibrosis in the intestine develops are incompletely understood. Therefore, insight in the pathogenesis of intestinal fibrosis is an unmet medical need in IBD care.

Over the last few decades, most studies on IBD have focused on the identification of abnormal adaptive immune responses. More recently, the focus has shifted toward mucosal innate immune responses. Recent data suggested a causal link between defects in the resolution of intestinal inflammation and impaired bacterial clearance, excessive cytokine secretion, altered monocyte‐macrophage transition, and activation of tissue innate immune cells.^6^, ^7^ Intestinal fibrosis is believed to be a chronic and progressive process triggered by inflammation through complex matrix/cell/protein interactions, but may be reversible.^8^

Innate lymphoid cells (ILC) were discovered as an additional source of mucosal cytokines.^9^, ^10^ Three groups of ILC have been defined based on shared expression of surface markers, transcription factors, and effector cytokines with T helper (Th) cell subtypes. Group 1 ILC (ILC1) are implicated in immunity to intracellular pathogens, influenced by interleukin 12 (IL‐12), they express T‐bet, and produce interferon γ (IFN‐γ) and tumor necrosis factor (TNF). Group 2 ILC (ILC2) are involved in type 2 inflammation required for antihelminth defenses and allergic inflammation, influenced by IL‐25, IL‐33, and TSLP, they express GATA‐3, and produce type 2 effector cytokines IL‐4, IL‐5, IL‐9, IL‐13, and amphiregulin. Group 3 ILC (ILC3) are involved in antibacterial immunity, chronic inflammation, and tissue repair, influenced by IL‐1β, IL‐6, and IL‐23, they express RORγt, and produce IL‐17 and/or IL‐22.^11^

ILC residing in the gut mucosa are important regulators of homeostasis and have the potential to control tissue remodeling.^12^, ^13^, ^14^ The role of ILC in mucosal immune pathology is not clear. In murine models of asthma, ILC2 are a source of IL‐4, IL‐5, and IL‐13 after stimulation with epithelial cell derived IL‐25 and IL‐33.^15^ In murine models of IBD, ILC contribute to the development and persistence of intestinal inflammation by production of IL‐17A and IFN‐γ in response to IL‐23.^16^ ILC3 are important players in protecting the epithelial barrier by production of IL‐22 in response to epithelial stress, however IL‐17 production by these cells can contribute to development of colitis.^17^, ^18^ Bernink et al showed that IFN‐γ producing ILC1 are increased in resected tissues of patients with CD, suggesting their involvement in human disease.^19^, ^20^ ILC might also play an important role in organ fibrosis. In murine models of lung and liver fibrosis, type 2 ILC have been shown to be essential for the development of fibrosis, via an IL‐ 25 and IL‐33 dependent pathway, respectively.^12^, ^14^

Previously, we and others have shown that chronic dextran sodium sulfate (DSS) colitis induced by repeated cycles of DSS exposure is characterized by transmural inflammation and granuloma formation and followed by remodeling and fibrosis.^21^, ^22^ To dissect the involvement of ILC in this process, chronic DSS colitis was induced in wild type (WT), in recombination activating gene (RAG^−/−^) deficient mice (which lack adaptive immunity but have a normal ILC population), in anti‐CD90.2 injected RAG^−/−^ mice and in RAG^−/−^ common γ chain^−/−^ (RAG^−/−^γc^−/−^) mice which both also lack the ILC population. This enabled us to separately analyse whether adaptive immunity and/or ILC are involved in colitis and fibrosis induction.

## 2 MATERIALS AND METHODS

### 2.1 Mice and induction of colitis

**Figure 1:**
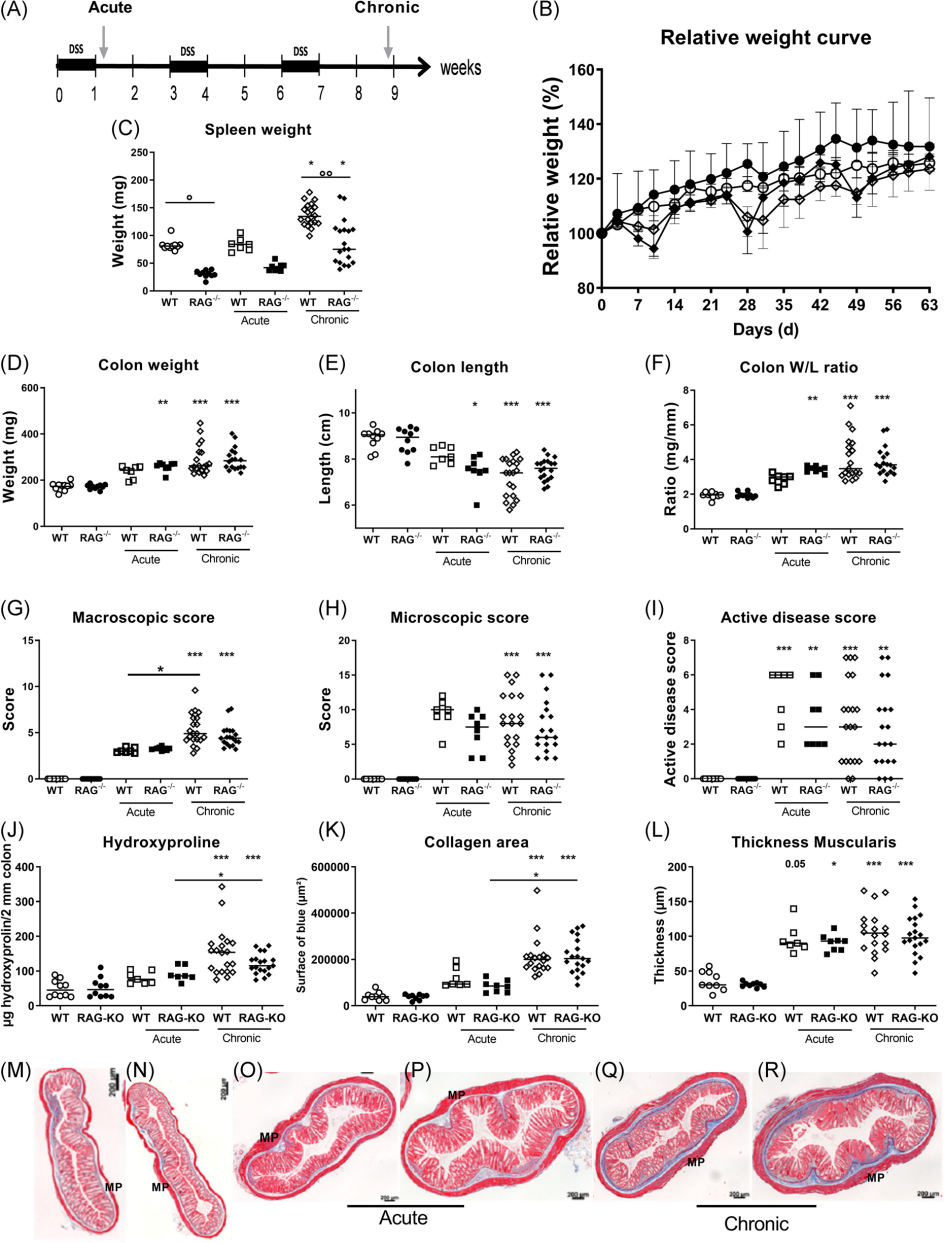
Induction of acute and chronic colitis with DSS in RAG^−/−^ as compared with WT mice. A, Study set‐up. Colitis was induced with DSS in drinking water. Acute colitis was induced in WT and RAG^−/−^ mice (*n* = 7 and 8) by 7 days of DSS administration without recovery. For induction of chronic colitis, WT and RAG^−/−^ mice (*n* = 20 and 19) were exposed to repeated cycles of DSS exposure. One cycle of DSS comprises 7 days of DSS in drinking water, followed by a recovery period of 14 days with normal drinking water. Inflammatory and fibrotic parameters were compared between both groups of DSS exposed mice, and also to control WT and RAG^−/−^ mice without DSS administration (*n* = 10 and 10). B, Relative weight curve of WT and RAG^−/−^ control mice and three cycles (chronic) of DSS. C, Spleen weight, (D) colon weight, (E) colon length, (F) colon W/L ratio, (G) macroscopic score, (H) microscopic score, and (I) histological active disease score. Fibrosis parameters: (J) hydroxyproline quantification, (K) collagen area, and (L) thickness of MP. Representative pictures of MSB staining indicating deposition of collagen (blue) in WT (M) and RAG^−/−^ (N) control mice, after one (acute) (O,P) and after three cycles (chronic) of DSS in WT (Q) and RAG^−/−^ (R) mice. Mice were age‐matched at time of sacrifice. Multiple comparison with Dunn's correction between groups; multiple comparison with Dunn's correction with background controls is shown above each group; *P < .05, **P < .01, ***P ≤ .001. Data are shown as individual values with median. Data are pooled from two independent experiments. DSS, dextran sulfate sodium; MP, muscularis propria; MSB, Martius Scarlet Blue; RAG, recombination activating gene; W/L, weight/length; WT, wild type

**Figure 2:**
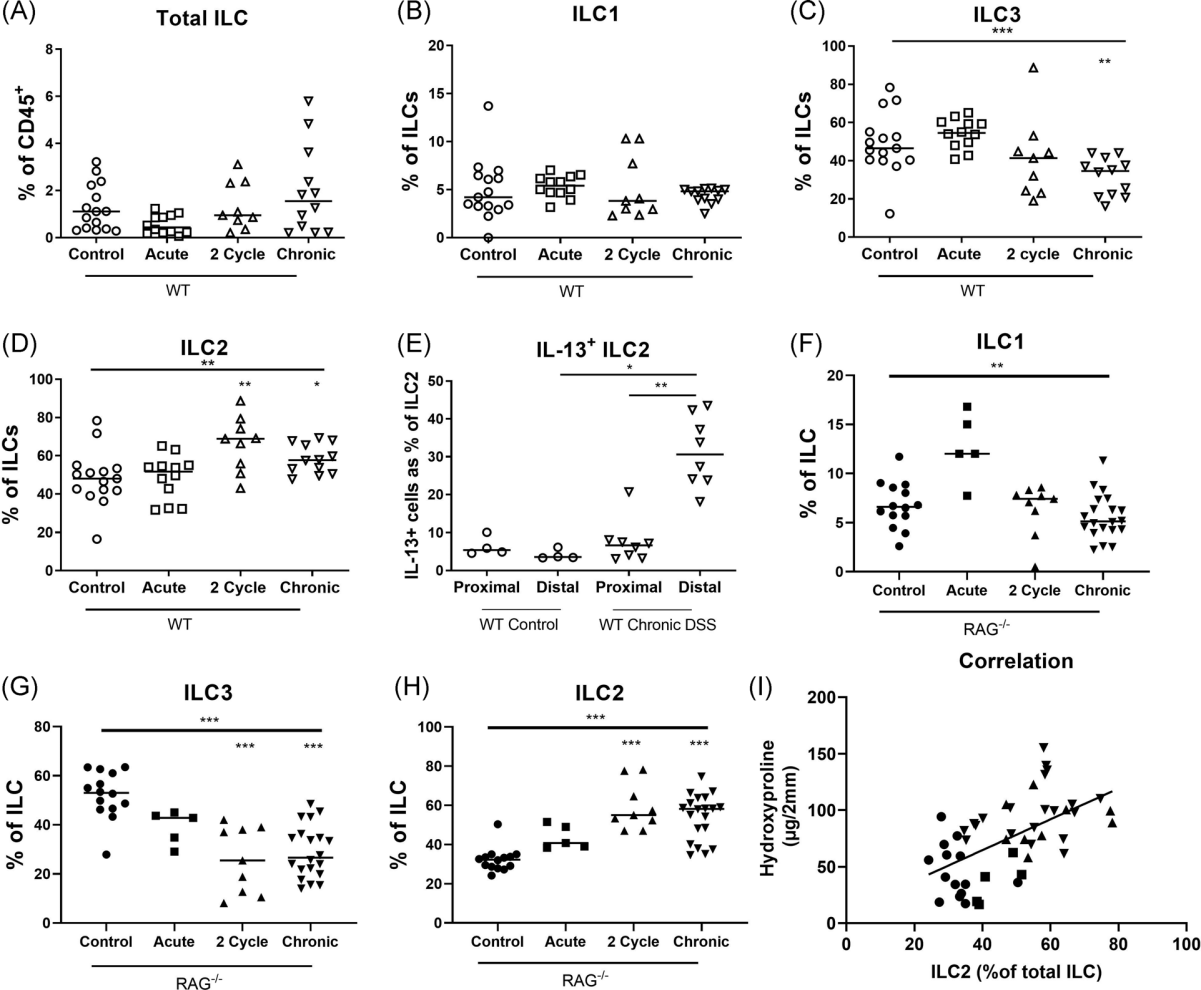
Levels of intestinal ILC2 during chronic remodeling in WT and RAG^−/−^ mice acute colitis, and two and three cycle chronic DSS colitis were induced in WT and RAG^−/−^ mice as explained in Figure 1. Lamina propria lymphocytes of the distal colon were stained for identification of ILC subtypes. Percentage of intestinal total ILC (A), ILC1 (B), ILC3 (C), and ILC2 (D) in WT mice with acute (*n* = 12), two cycle (*n* = 9), and chronic (three cycle, *n* = 12) DSS colitis as compared with WT controls (*n* = 15). E, Percentage of IL‐13 positive ILC2 in proximal and distal colon after in vitro PMA/ionomycin stimulation after three cycles of DSS as compared with control mice without DSS administration. Percentage of intestinal ILC1 (F), ILC3 (G), and ILC1 (H) in RAG^−/−^ mice with acute (*n* = 5), two cycle (*n* = 9), and chronic (three cycle, *n* = 20) DSS colitis as compared with RAG^−/−^ controls (*n* = 14). Correlation of ILC2 with hydroxyproline levels in colon of RAG^−/−^ mice with chronic three cycle DSS colitis (I). Data are shown as individual values and median. Results of Kruskal‐Wallis testing is shown on top in each figure, significance of results of multiple comparison with Dunn's correction as compared with controls is shown above each group Spearman correlation (E). *P < .05, **P ≤ .01, ***P ≤ .001. Data are pooled from three independent experiments. DSS, dextran sulfate sodium; IL, interleukin; ILC, innate lymphoid cell; PMA, phorbol myristate acetate; RAG, recombination activating gene; WT, wild type

**Figure 3:**
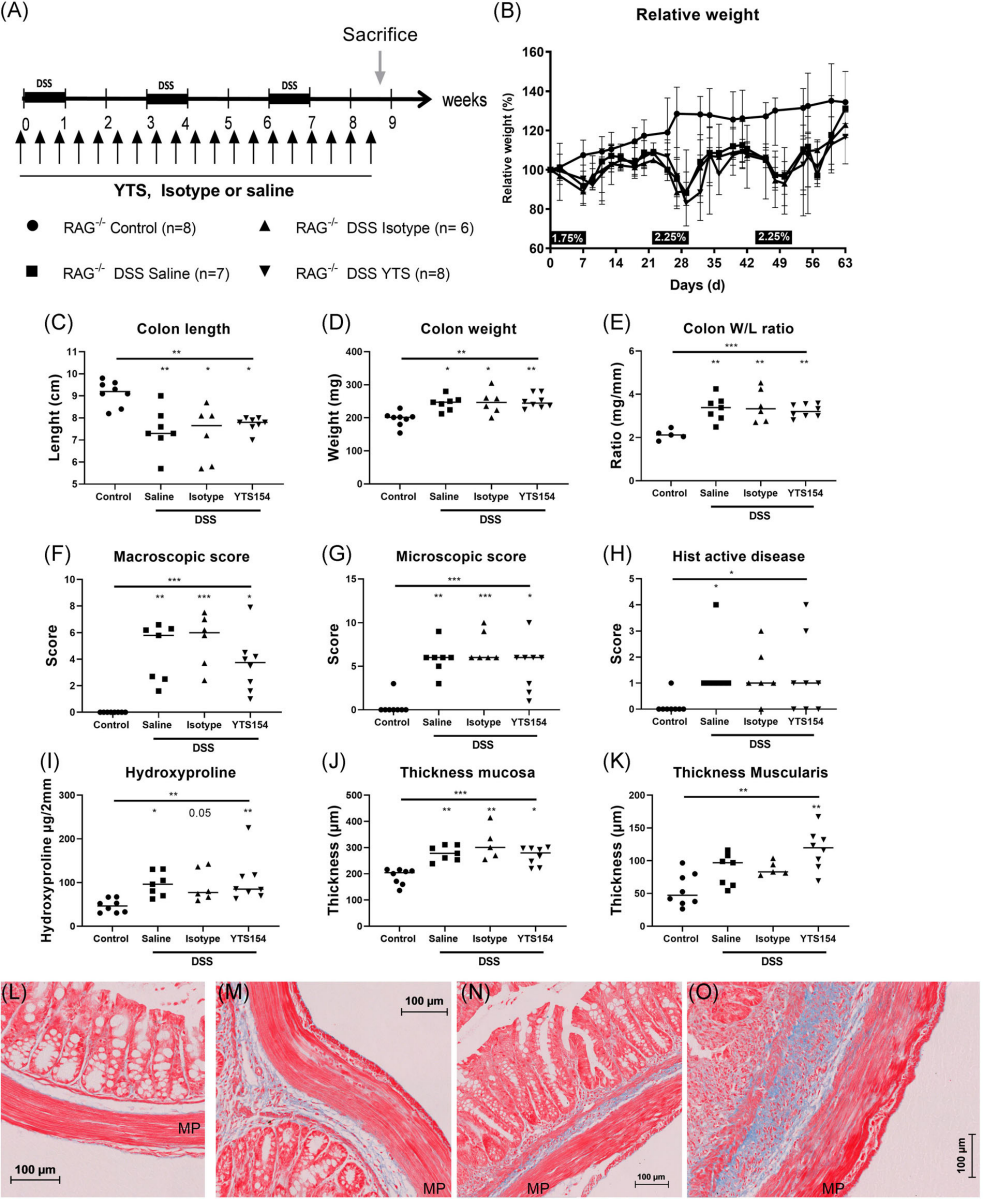
Effect of ILC depletion with monoclonal antibody to CD90.2 on chronic DSS in RAG‐1^−/−^ mice. A, RAG^−/−^ were injected every 3 days with saline (*n* = 7), isotype (*n* = 6), or anti‐CD90.2 (YTS) antibody (*n* = 8) and exposed to three cycles of DSS and compared with control mice without DSS administration (*n* = 8). B, Relative weight curve. Colon parameters: length (C), weight (D), and W/L ratio (E). Disease activity markers; macroscopic score (F), microscopic score (G), and histological active disease score (H). Representative MSB stainings (blue = collagen) of control mice without DSS administration (L) and chronic DSS colitis mice treated with saline (M), isotype (N), and YTS (O) injections. Results of Kruskal‐Wallis testing is shown on top of each figure, significance of results of multiple testing with Dunn's correction compared with healthy controls is shown above the group; *P < .05, **P ≤ .01, ***P ≤ .001. Data are shown as individual values with median. Data are pooled from two independent experiments. DSS, dextran sulfate sodium; MP, muscularis propria; MSB, Martius Scarlet Blue; RAG, recombination activating gene; W/L, weight/length

**Figure 4:**
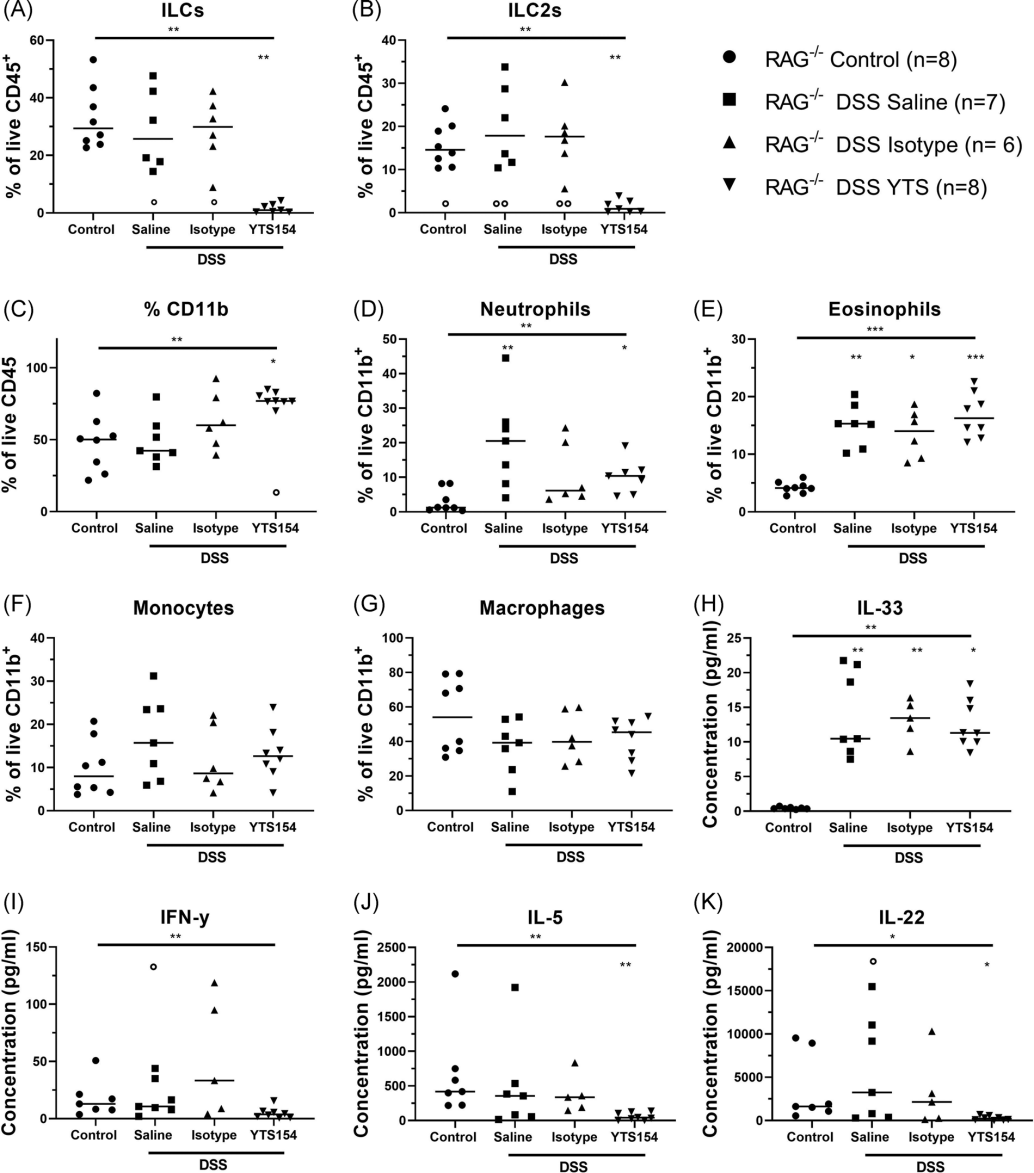
Effect of anti‐CD90.2 injections on leukocyte populations and cytokines in distal colon of RAG‐1^−/−^ with chronic DSS colitis RAG^−/−^ were injected with saline (*n* = 7), isotype (*n* = 6), or YTS antibody (*n* = 8) and exposed to three cycles of DSS and compared with control mice without DSS administration (*n* = 8) as detailed in the legend to Figure 3. Colonic lamina propria immune cells were isolated, and the different types of innate immune cells were identified by staining for specific markers and flow cytometry. Relative contribution of (A) innate lymphoid cells, (B) ILC2, and (C) CD11b+ cells within the total CD45+ population. Relative contribution of (D) neutrophils, (E) eosinophils, (F) monocytes, and (G) macrophages in the CD11b+ population. Proteins secreted by the cells from the affected distal colon were quantified after a 24 hours ex vivo culture. Levels of (H) IL‐33, (I) IFN‐y, (J) IL‐5, and (K) IL‐22. Results of Kruskal‐wallis testing is shown on top of each figure, significance of results of multiple testing with Dunn's correction compared with control mice without DSS exposure is shown by * above the group; significance of results of multiple testing with Dunn's correction compared with YTS treated mice is shown as ° above the group. *P < .05, **P ≤ .01, ***P ≤ .001. Data are shown as individual values with median. Data are pooled from two independent experiments. DSS, dextran sulfate sodium; IFN, interferon; IL, interleukin; ILC, innate lymphoid cell; RAG, recombination activating gene

**Figure 5:**
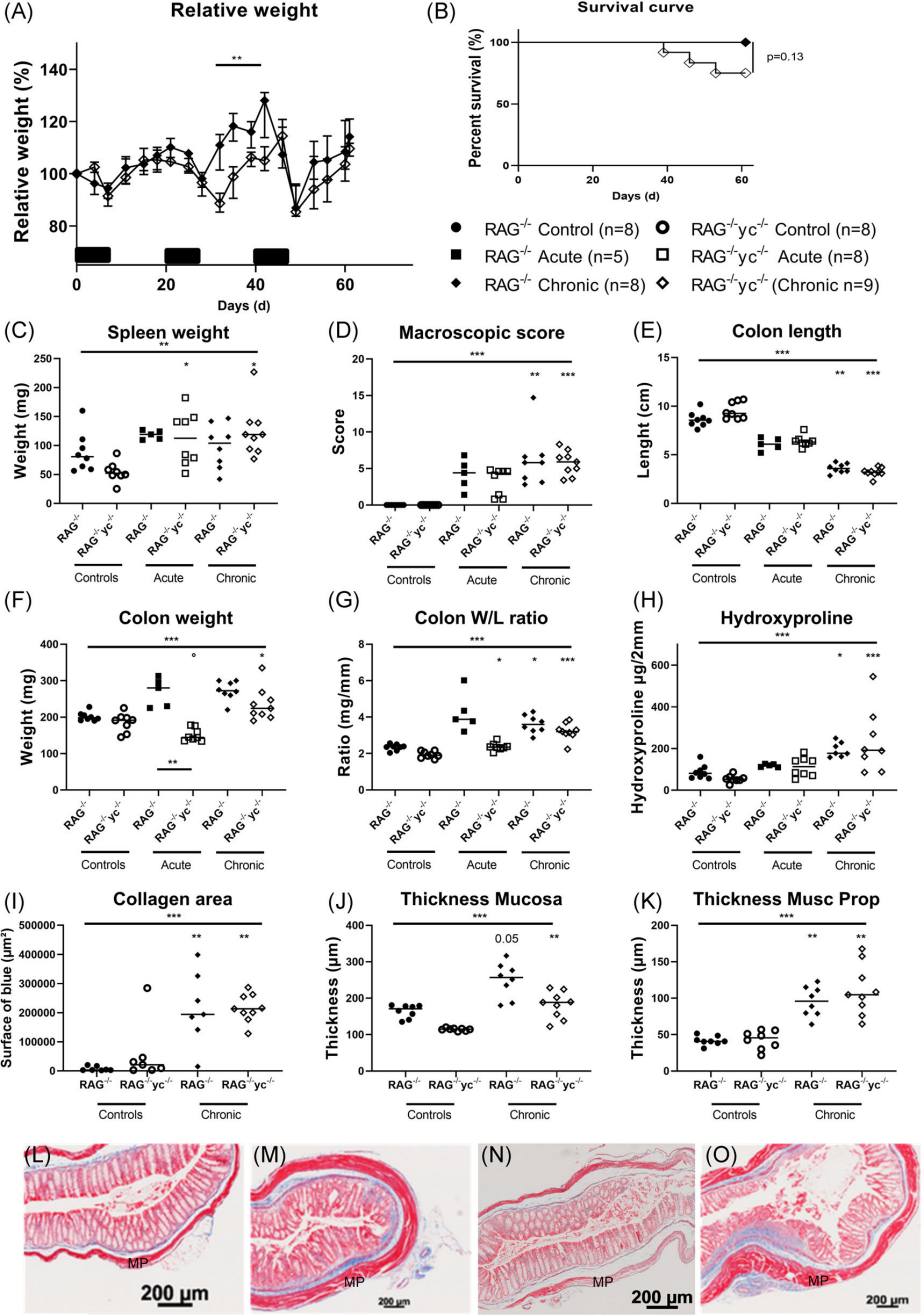
Fibrosis is not attenuated in RAG^−/−^γc^−/−^ mice after three cycles of DSS. RAG^−/−^ and RAG^−/−^γc^−/−^ mice were cohoused during either acute (RAG^−/−^: *n* = 5 and RAG^−/−^γc^−/−^: *n* = 8) or chronic DSS colitis (RAG^−/−^: *n* = 8 and RAG^−/−^γc^−/−^: *n* = 9) compared with control mice without DSS exposure (*n* = 8 for both strains) and killed on day 9 (acute) or day 63 (chronic). Relative weight curve (A) and Kaplan‐Meier curve (B) (only RAG^−/−^ and RAG^−/−^γc^−/−^ chronic DSS shown to highlight discrepancies). Disease parameters: spleen weight (C), macroscopic score (D), colon length (E), colon weight (F), and colon W/L ratio (G). Fibrosis parameters: hydroxyproline quantification (H), quantification of the surface of collagen in MSB staining (I), thickness of the mucosa (J), and thickness of the muscularis propria (K). Representative pictures of MSB staining in RAG^−/−^ control mice without DSS exposure (L) and chronic DSS colitis mice (M) compared with RAG^−/−^γc^−/−^ control (N) and chronic DSS colitis mice (O). Kruskal‐wallis testing is shown, multiple testing with Dunn's correction compared with healthy background controls is shown above each group; multiple testing with Dunn's correction between groups is shown by a connecting line. *P < .05, **P ≤ .01, ***P ≤ .001. Data are shown as individual values with median. Data are pooled from two independent experiments. DSS, dextran sulfate sodium; MP, muscularis propria; MSB, Martius Scarlet Blue; RAG, recombination activating gene; W/L, weight/length

**Figure 6:**
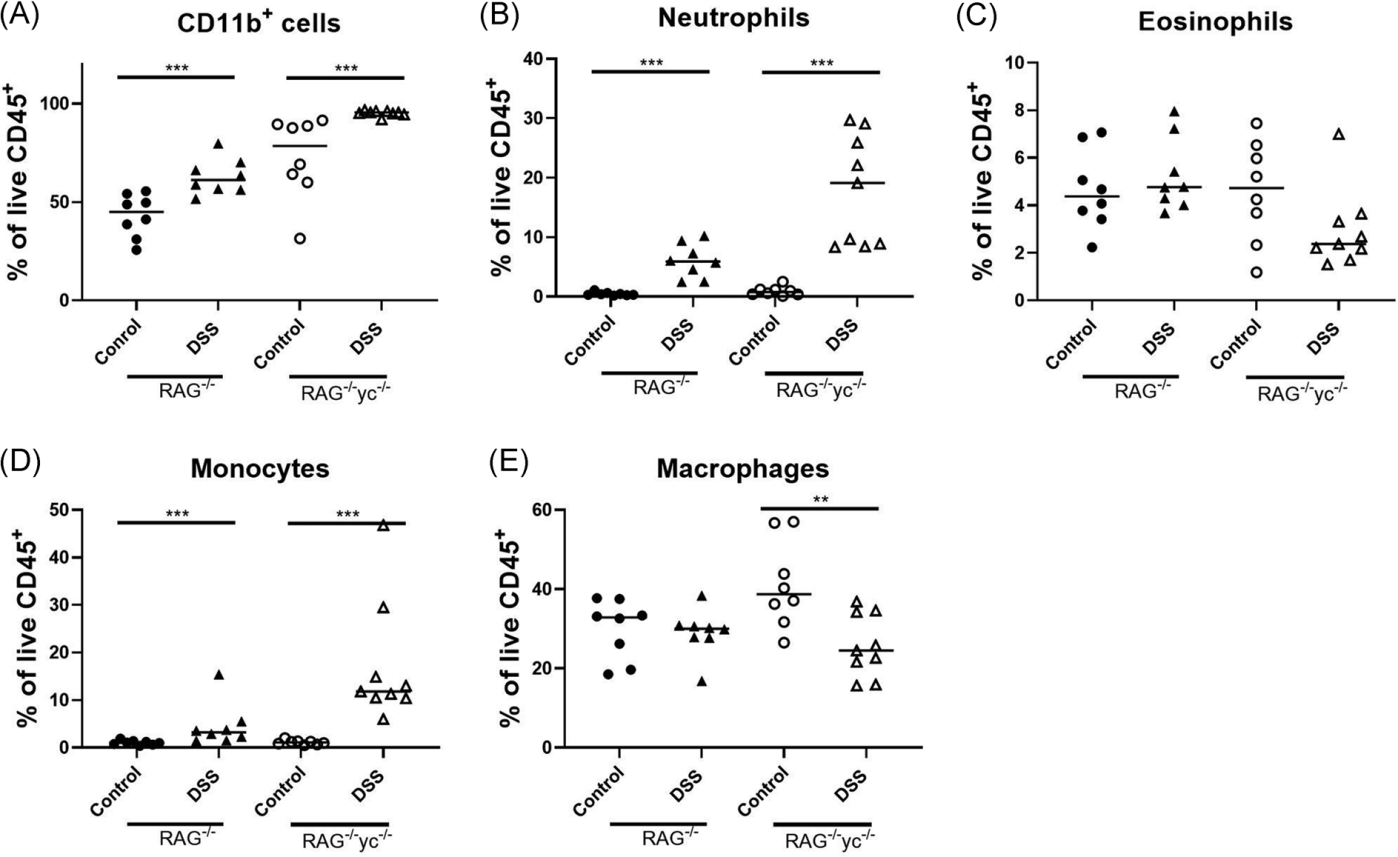
Infiltrating myeloid cells after induction of chronic colitis, RAG^−/−^ and RAG^−/−^γc^−/−^ mice were cohoused during either acute (RAG^−/−^: *n* = 5 and RAG^−/−^γc^−/−^: *n* = 8) or chronic DSS colitis (RAG^−/−^: *n* = 8 and RAG^−/−^γc^−/−^: *n* = 9) and compared with control mice without DSS administration (*n* = 8 for both). Mice were killed on day 9 (acute) or day 63 (chronic). Colonic lamina propria cells were isolated, and the different types of myeloid cells were identified by staining and flow cytometry. Relative contribution of (A) total CD11b+ cells, (B) neutrophils, (C) eosinophils, (D) monocytes, and (E) macrophages as % of the total CD45+ population. Results of Mann‐Whitney *U* testing to compare control and DSS groups within each background is shown. *P < .05, **P ≤ .01, ***P ≤ .001. Data are shown as individual values with median. Data are pooled from two independent experiments. DSS, dextran sulfate sodium; RAG, recombination activating gene

To induce DSS colitis, female 6‐week‐old C57BL/6OlaH WT mice were obtained from Envigo (Horst, The Netherlands). RAG1^−/−^^tm/tm^ mice, lacking T and B cells, were obtained from Charles River (MA) (mice in figure 1 and 2) or were bred in our own animal facility (figures 3‐8).^21^ Female 6‐week‐old C57BL/6NTac;B10(Cg)‐Rag2^tm1Fwa^ Il2rg^tm1Wjl^ (RAGγc^−/−^) mice were obtained from Taconic (New York). All animals were maintained in the Animal Care Facility of the Faculty of Medicine, University of Leuven (Belgium) and according to ARRIVE guideline. Colitis was induced with DSS as previously described.^20^ Briefly, 1.5% to 2.25% DSS (35‐50 kDa; MP Biomedicals, Illkirch, France) was added to the drinking water to induce colitis. Acute colitis mice received DSS for 7 days. For the study of chronic colitis, mice were exposed to repeated “cycles” of DSS exposure. One cycle was defined as exposure to DSS for 7 days followed by a recovery period of 2 weeks with normal drinking water (Figure 1A). Mice were exposed to one, two, or three cycles in total. All mice were age‐matched at the time of sacrifice.

### 2.2 Evaluation of inflammation and fibrosis

Animals were euthanized with sodium pentobarbital (Nembutal; Ovation Pharmaceuticals Inc, Deerfield, IL) and Disease Activity Index (DAI) was determined based on body weight loss, stool consistency, and presence of gross blood in the stools. Next, inflammation in distal colon was evaluated as previously described.^18^ A macroscopic damage score was calculated based on the presence of adhesions, hyperemia, and extent of colonic inflammation. Paraffin embedded, 5 µm‐thick longitudinal and transverse sections were stained with hematoxylin and eosin. The microscopic score of inflammation was calculated as previously described.^18^ Briefly, this score comprised the sum of changes in mucosal architecture, neutrophil infiltration, mononuclear cell infiltration, goblet cell loss, and epithelial defects. The histological active disease score comprised the sum of neutrophil infiltration and epithelial defects, reflecting acute tissue damage during the last 24 hours before tissue sampling. Tissue sections were scored by an experienced pathologist (GdH) blinded to the experimental conditions. Fibrosis was also evaluated by hydroxyproline quantification and Martius Scarlet Blue (MSB) stainings as previously described.^20^, ^23^ In short, areas stained for collagen in the mucosa and submucosa were measured in two cross‐section using ImageJ.^20^ The thickness of the mucosa and muscularis propria was calculated as mean value of two different measurements per mouse on uniform horizontal cross sections of colon crypts using ImageJ.^22^, ^24^ The hydroxyproline assay was performed as previously described to quantify the amount of collagen.^25^

### 2.3 Antibody‐mediated depletion of ILC

YTS154 (rat anti‐mouse CD90.2, cell clone provided by H. Waldmann, University of Cambridge, Cambridge, England, UK), to deplete CD90.2 expressing ILC, was produced at Bioceros and was used as previously described.^26^ Rat IgG1 isotype (clone MCP‐11) was obtained from BioXCell (West Lebanon, NH). Antibodies or corresponding isotype controls, diluted in sterile phosphate‐buffered saline (PBS), were administered intraperitoneally every 3 days (250 µg/mouse) starting from 3 days before DSS exposure and during the whole experiment (from day 3 until day 63). In control mice, not exposed to DSS, the corresponding volume of sterile PBS was administered.

### 2.4 Cell isolation and flow cytometry

Distal colon sections were cut longitudinally and kept in complete medium (RPMI‐1640; Lonza, Belgium) supplemented with 10% FBS (Thermo Fisher Scientific, Belgium) and 1% antibiotic‐antimyotic 100× (Thermo Fisher Scientific). To remove mucus and epithelial cells, samples were incubated twice under magnetic stirring for 20 minutes at 37°C in complete medium supplemented with 5 mM dithiothreitol (Chem‐Lab, Belgium) and 5 mM EDTA (Lonza). The epithelial fraction was discarded, and remaining tissue was digested for 40 minutes at 37°C under magnetic stirring in Hank's balanced salt solution with Ca^2+/^Mg^2+^ (Lonza) supplemented with 3 mg/mL dispase (Gibco, Japan), 1 mg/mL collagenase D (Worthington), 0.2% DNase I (Roche, Germany), and 2% HEPES (Thermo Fisher Scientific). Lamina propria lymphocytes were filtered through a 70 µm cell strainer (Greiner Bio‐One, Belgium) and incubated for 30 minutes in fixable viability dye 780 (FVD780; Thermo Fisher Scientific) at room temperature before staining with antibodies listed in Table S1 and Figure S1 to S3. ILC were gated as CD45^+^Lineage^−^ (Lin: CD3e, CD5, CD11b, CD19, CD94, TCRγδ, and Ter‐119) CD127^+^CD90.2^+^ cells. Within the ILC, ILC3 were defined as RORyt^+^, ILC2 were defined as Killer cell lectin‐like receptor subfamily G member (KLRG‐1)^+^RORyt^−^/dim and ILC1 were defined as KLRG‐1^−^RORyt^−^NK1.1^+^ cells. Myeloid cell types were defined based on the following markers: neutrophils (CD45^+^CD11b^+^Ly6G^+^), eosinophils (CD45^+^CD11b^+^ Siglec‐8^+^), mast cells (CD45^+^CD11b^−^CD117^+^Fcεr^+^), macrophages (CD45^+^CD11b^+^CD64^+^MHCII^+^), and monocytes (CD45^+^CD11b^+^Ly6C^+^). Intracellular staining of RORγt was performed according to manufacturing instructions of the Foxp3 staining kit (Invitrogen). For intracellular cytokine detection, cells were stimulated with phorbol myristate acetate 100 ng/mL and ionomycin 1 µg/mL (Sigma‐Aldrich) for 6 hours and monensin 0.7 µg/mL (Sigma‐Aldrich) for 4 hours followed by anti‐IL13 staining. After staining, cells were fixated in 1% paraformaldehyde.

Flow cytometry was conducted on a BD LSR Fortessa instrument in accordance with standard methods. Calibration was performed before each acquisition by CS&T beads (BD). For fluorescence compensation settings, single color UltraComp eBeads compensation beads were used (Thermo Fisher Scientific). Fluorescence Minus One controls were included.

### 2.5 Protein quantification

A 0.5 cm piece of affected distal colon was opened longitudinal and incubated for 24 hours at 37°C in complete medium before supernatant collection. Protein concentrations in the supernatants for interleukin 4 (IL‐4), IL‐5, IL‐6, IL‐12p70, IL‐22, IL‐23p19, TNF, and IFN‐γ were determined using multiplex meso scale discovery technologies. In short, samples were incubated overnight at 4°C on a U‐plex plate coated with biotinylated capture antibodies. After three wash steps in PBS 0.05% tween (Sigma‐Aldrich), detection antibodies were added for 1 hour at room temperature before activation of the plate electrodes resulting in a quantitative measurable emission of light.

### 2.6 Statistical analysis

Statistical analysis were performed using GraphPad Prism 8 (GraphPad, La Jolla, CA). Data are represented as medians (interquartile range) and the individual *P* values for two group comparison were obtained using Mann‐Whitney *U* testing. The following methods were used: multiple comparison with Dunn's correction between multiple groups, Spearman for correlation testing, and Kaplan‐Meier curve for survival analysis. Differences were considered statistically significant at *P < .05, **P ≤ .01, and ***P ≤ .001.

### 2.7 Ethical considerations

All studies were approved by and performed according to the local ethics committee for animal experimentation of the University of Leuven (P230‐2015).

## 3 RESULTS

### 3.1 Inflammation in acute and chronic colitis is similar in WT and RAG‐1^−/−^ mice

To study whether adaptive immunity is required for development of colitis in the DSS model, we compared WT mice with RAG‐1^−/−^ mice lacking B and T lymphocytes.^27^ Acute DSS colitis was induced by DSS ingestion through drinking water (Figure 1A). Weight loss after 1 week was more pronounced in the RAG‐1^−/−^ mice than in WT mice (*P* = .006; Figure 1B). Spleen weight did not increase after 1 week of DSS exposure (*P* > .99 for both backgrounds; Figure 1C). DAI scores were significantly higher in RAG‐1^−/−^ mice exposed to DSS compared with DSS exposed WT mice (*P* = .026; *data not shown*). Colon length decreased in both strains after 1 week of DSS exposure (WT: *P* = .006 and RAG‐1^−/−^: *P* = .001), but significantly more in RAG‐1^−/−^ mice compared with WT mice (*P* = .032; Figure 1D,E), resulting in a significantly higher colon weight/length (W/L) ratio in RAG‐1^−/−^ mice compared with WT mice (*P* < .001; Figure 1F). No significant differences could be observed in macroscopic damage score, microscopic score of inflammation, and histological active disease score between RAG‐1^−/−^ mice and WT mice (Figure 1G‐I).

For chronic colitis, we used three cycles of DSS exposure in RAG‐1^−/−^ and WT mice. One cycle of DSS exposure consisted of 1 week of DSS exposure followed by a recovery period of 2 weeks. RAG‐1^−/−^ mice lost more weight during three cycles of DSS administration than WT mice (Figure 1B). Spleen weight, colon length, colon weight, colon W/L ration, macroscopic damage score, microscopic score of inflammation, and histological active disease score were similar in RAG‐1^−/−^ mice compared with WT mice in this chronic model (Figure 1D‐I).

We also analysed the composition of inflammatory cells in the colonic mucosa. Gating of the different myeloid cell populations is illustrated in Figure S1. When compared with controls without DSS exposure, neutrophils (14.40% vs 0.63% of CD11b^+^ cells, *P* = .004) and monocytes (28.40% vs 8.16% of CD11b^+^ cells *P* = .004) were increased in WT mice after three cycles of DSS (Figure S2). In contrast, decreased proportions of eosinophils (18.30% vs 7.94% CD11b^+^ cells, *P* = .016) and macrophages (57.80% vs 33.51% CD11b^+^ cells %, *P* = .004) were observed after three cycles of DSS in WT mice (Figures S2B and S2D). Similarly, in RAG‐1^−/−^ mice with chronic DSS colitis, neutrophils (9.50% vs 0.61% of CD11b^+^ cells, *P* = .004) and monocytes (5.78% vs 1.78% of CD11b^+^ cells, *P* = .028) were increased as compared with RAG‐1^−/−^ mice without DSS exposure, while no significant effect was seen on eosinophils and macrophages (Figure S2E‐H).

In summary, after 1 week of DSS exposure (acute model) or after three cycles of DSS administration (chronic model), systemic and colonic inflammation were similar or slightly more severe in RAG‐1^−/−^ mice lacking T and B lymphocytes vs WT mice.

### 3.2 Intestinal fibrosis is induced despite the absence of adaptive immunity

Intestinal fibrosis was then evaluated in the acute and chronic colitis models. Collagen deposition was clearly more pronounced in chronic three‐cycles DSS colitis compared with acute colitis, in both WT and RAG‐1^−/−^ mice as shown by MSB collagen quantification (*P* = .003 and *P* < .001, respectively) and hydroxyproline measurements (*P* = .003 and *P* < .001, respectively; Figure 1J‐R). However, both fibrosis quantification methods revealed similar collagen deposition in RAG‐1^−/−^ mice versus WT mice in the chronic model (*P* > .999 for both; Figure 1J,K). Notably, no differences were observed with regard to the thickness of the muscularis propria (Figure 1L). Altogether, these results suggest that the adaptive immunity is not required for the intestinal fibrogenesis process in chronic DSS colitis. We, therefore, turned our attention to a potential involvement of ILC.

### 3.3 Increased IL‐13 producing ILC2 after two and three cycles of DSS in WT and RAG‐1^−/−^ mice

To study whether ILC are involved in intestinal fibrosis in this model we first studied the distribution of mucosal ILC subsets during acute or repetitive DSS administration in WT mice. The gating strategy for ILC and the identification of subsets based on membrane markers and intracellular RORγt expression are shown in Figure S3. No changes were observed in the total ILC proportion in acute colitis or after two and three cycles of DSS exposure (Figure 2A). The percentage of ILC1 was not different after 1 week of DSS exposure as compared with control mice without DSS exposure (5.42% vs 4.21% of ILC, *P* > .999; Figure 2B). However, after two or three cycles of DSS in WT mice, we observed a decrease of ILC3 and a relative increase in KLRG‐1^+^ ILC2 in the colon mucosa as compared with control mice without DSS exposure (ILC2: 68.90% and 57.70% vs 48.00% of ILC, *P* = .012 and *P *= .023; Figure 2C,D). Among these ILC2, an increased proportion expressed IL‐13 in the chronic model as compared with control mice (30.60% vs 6.60% of ILC2, *P* = .004; Figure 2E). However, the mRNA levels of *IL‐13* in total colon tissue at sacrifice were not increased (data not shown).

In RAG‐1^−/−^ mice, ILC represent 27.55% of CD45^+^ leukocytes in the colonic lamina propria. After two and three cycles of DSS, the increase in KLRG‐1^+^ ILC2 was confirmed in RAG‐1^−/−^ mice as compared with RAG‐1^−/−^ mice without DSS administration (55.50% and 58.20% vs 32.30% of ILC, both *P* < .001; Figure 2F‐H). Notably, ILC2 proportions among ILC correlated with hydroxyproline quantification (*r* = .61, *P* < .001) suggesting a possible relation between ILC2 and fibrosis (Figure 2I).

### 3.4 ILC are not crucial for induction of fibrosis in chronic DSS

To further investigate the contribution of ILC to colitis and fibrosis, ILC were depleted in the chronic DSS model in RAG‐1^−/−^ mice using YTS, an anti‐Thy1.2 (CD90.2) depleting monoclonal antibody (mAB). To exclude effects of antibody treatment on CD90.2 expressing epithelial cells and fibroblasts, control RAG‐1^−/−^ mice (not exposed to DSS) were injected at a similar dosing schedule.^25^ We did not see any macroscopic or microscopic effect on the colon architecture as a result of treatment (Figure S4C,D).

Efficacy of ILC depletion by anti‐CD90.2 (YTS) injection was confirmed by a reduction of ILC in the colon as compared with control mice and saline or isotype injected chronic DSS colitis mice (0.99% vs 29.35%, 25.70%, and 29.85% of CD45^+^ cells, *P* = .002, *P *= .029, and *P* = .018, respectively; Figure 4A and S4A,B). We also confirmed that YTS injection resulted in ILC2 depletion (YTS: 0.90 vs saline: 14.59%, isotype: 17.84%, and controls: 17.65% of CD45^+^, *P* = .015, *P *= .006, and *P *= .010; Figure 4B).

After elimination of ILC and chronic DSS exposure, weight loss was similar as compared with isotype and saline treated RAG‐1^−/−^ mice with chronic DSS colitis (Figure 3A,B). At sacrifice, colon length, colon weight, colon W/L ratio, macroscopic damage score, microscopic score of inflammation, and histological active disease score were not different after ILC depletion as compared with chronic DSS controls (Figure 3C‐H and S4 C‐SG). Hydroxyproline levels and thickness of muscularis propria and mucosa of the distal colon (which in this model is the most affected part) were not altered after ILC depletion as compared with saline or isotype injected chronic DSS colitis controls (Figure 3I‐O). We also studied the inflammatory cells in the LP cell suspension isolated from the colonic mucosa. ILC depletion resulted in higher proportion of CD11b^+^ myeloid cells as compared with saline treated chronic DSS colitis controls (76.90% vs 42.40% of CD45^+^, *P* = .041; Figure 4C). Within the myeloid population, we found an increase of neutrophils (YTS: 10.40, saline: 20.50 vs control: 1.25% of CD11b^+^ cells, *P* = .041 and *P *= .004) and eosinophils (YTS: 16.25%, saline: 15.30%, isotype: 14.00% vs control: 4.14% of CD11b^+^cells, *P* = .001, *P *= .008, and *P *= .042) after chronic DSS as compared with mice without DSS exposure (Figure 4D,E), but no differences were observed related to treatment. Also, no differences in levels of monocytes and macrophages were observed in the different groups (Figure 4F,G).

Antibody‐mediated ILC depletion resulted in a lower production of ILC derived cytokines IL‐5 (39.74 vs 418.60 pg/mL, *P* = .002) and IL‐22 (218.30 vs 1619.00 pg/mL, *P* = .046) and a tendency toward lower production of IFN‐γ (3.81 vs 12.92 pg/mL, *P* = .15), as compared with DSS exposed control groups (Figure 4I‐K). In contrast, levels of IL‐33, an epithelial cell derived cytokine, were elevated during chronic DSS exposure and were elevated in absence of ILC as compared with DSS unexposed control mice (10.47 and 11.31 vs 0.37 pg/mL, *P* = .013; Figure 4H).

### 3.5 ILC deficient mice recover more slowly after chronic inflammatory insult but have similar inflammation and fibrosis on DSS exposure

We validated the anti‐CD90.2 mediated ILC depletion findings in an ILC‐deficient RAG^−/−^γc^−/−^ background. RAG^−/−^γc^−/−^ mice were cohoused with RAG‐1^−/−^ mice during induction of acute and chronic DSS colitis (Figure 5A‐O). In acute colitis, weight loss, spleen weight, and macroscopic damage score of the colon was similar in RAG^−/−^γc^−/−^ and RAG^−/−^ mice (Figure 5A‐D). In both strains there was a similar decrease in colon length (RAG‐1^−/−^γc^−/−^: 6.30 vs 9.25 cm, *P* < .001, RAG‐1^−/−^: 6.10 vs 8.55, *P* = .002), while a lower colon weight was observed in RAG^−/−^γc^−/−^ mice compared with RAG^−/−^ mice (RAG^−/−^γc^−/−^: 144.5 vs 191.5, *P* = .006, RAG^−/−^: 280.0 vs 198.0 mg, *P* = .003; Figure 5E‐G). Importantly, after the second cycle of DSS, slower recovery of weight was seen in RAG^−/−^γc^−/−^ mice as compared with RAG‐1^−/−^ mice (d31: 88.64% vs 111.0% of initial weight, *P* < .001; d35: 98.86% vs 118%, *P* = .001; d39: 106.3% vs 116%, *P* = .006 and d42: 105.0% vs 128.1%, *P* = .002; Figure 5A). Furthermore mortality was higher, but not significantly, in RAG^−/−^γc^−/−^ mice as compared with RAG‐1^−/−^ (*P* = .13; Figure 5B). After three cycles of DSS no differences in spleen weight or macroscopic colitis score were observed between both strains (Figure 5C,D). Although colon length decrease was slightly more pronounced in RAG^−/−^γc^−/−^ as compared with RAG‐1^−/−^ mice (7.15 vs 8.30 cm, *P* = .046), no differences in colon weight and colon W/L ratio were observed (Figure 5E‐G). Hydroxyproline levels and thickness of mucosa and muscularis propria were not different in RAG^−/−^γc^−/−^ as compared with RAG‐1^−/−^ mice with chronic DSS (Figure 5I‐O).

We characterized myeloid cells isolated from the colonic mucosa in RAG‐1^−/−^ and RAG^−/−^γc^−/−^ mice. After induction of chronic colitis, the increase in neutrophils (19.10% vs 5.91% of CD45^+^ cells, *P* = .004) and monocytes (11.80% vs 3.25% of CD45^+^ cells, *P* = .004) was more pronounced in RAG^−/−^γc^−/−^ mice as compared with RAG‐1^−/−^ mice (Figure 6B‐D). In contrast, eosinophils were not elevated in RAG^−/−^γc^−/−^ mice after chronic DSS administration (2.38% vs 4.70% of CD45^+^ cells, *P* = .114; Figure 6C).

## 4 DISCUSSION

This study intended to analyse the involvement of the adaptive immune system and of ILC in a model of chronic colitis, with special focus on fibrosis induction. As inflammation and fibrosis were unaltered in RAG‐1^−/−^ mice, adaptive immunity is clearly not required for the induction of fibrosis in this model. The redundancy of the adaptive immune system has previously been shown in acute DSS colitis.^26^, ^27^, ^28^, ^29^, ^30^ However, here we also show that in absence of the adaptive immune system there is not only unaltered induction of chronic inflammation, but also fibrosis can still be induced to the same extent as in the presence of T and B cells.

We then focused our attention on ILC as potential pathogenic cells. First, we could show that in chronic DSS colitis there is an increased proportion of ILC2 among ILC, both in absence and presence of the adaptive immune system. This was accompanied by a decrease in ILC3. Second, we obtained evidence for increased ILC activity upon DSS exposure, as ILC2 in the distal colon produced IL‐13 (a cytokine thought to be important for lung fibrogenesis), and as there was decreased production of IL‐5 and IL‐22 in the colonic mucosa of anti‐CD90 injected mice. As a relative shift from ILC3 predominance toward ILC2 predominance in the colon was observed both in WT and RAG^−/−^ mice, and as the manifestations of ILC2 expansion persist in RAG^−/−^ mice, this indicates that these changes in ILC activity and expansion occur independently of T cell activation. The most likely possibility is that they are the result of epithelial cell triggering and damage by DSS. We could indeed demonstrate that DSS exposure induces IL‐33 production, probably by epithelial cells, which is a potent inducer of ILC2 activity and can lead to the activation of the amphiregulin/EGFR pathway. This can potentially explain the expansion of ILC2 during intestinal remodeling in our model.^31^, ^32^ Importantly, the high IL‐33 production persisted in the absence of ILC after YTS treatment.

To know whether ILC contribute to tissue damage, fibrosis, and/or recovery in colitis, we then used a mAb to deplete ILC in RAG‐1^−/−^ mice. RAG‐1^−/−^ mice have a relative high proportion of ILC among CD45+ cells. To confirm the findings we also used a mouse strain lacking both ILC and adaptive immunity. Importantly, in both models no attenuation of fibrosis was observed neither after depletion of ILC nor in the absence of ILC, thus indicating that their role in fibrogenesis is negligible. However, in the absence of ILC a slower resolution of inflammation highlighted by a slower recovery of weight and higher mortality was observed rather pointing to a protective role of ILC.

In contrast to murine models of lung and liver fibrosis, in our model depletion of ILC thus did not result in an attenuation of fibrosis.^12^, ^14^ Although the ILC2 in the DSS model produced IL‐13 which is a profibrogenic cytokine, IL‐13 is not required for fibrosis. We indeed have previously shown that fibrosis in this model can be induced in IL‐13 deficient mice.^21^, ^33^ Our results are in contrast to the abrogation of acute and chronic innate colitis upon ILC depletion in an anti‐CD40 model.^16^ The anti‐CD40 model is characterized by strong activation of macrophages and dendritic cells which supposedly then interact directly with ILC.^34^ In DSS colitis, chemical disruption of the epithelial barrier might bypass the need of ILC by direct activation of fibroblasts. Further studies are required to identify differences in the pathways leading to fibroblast activation in both models. Furthermore, *Salmonella*‐induced infection models of lung and colon fibrosis are prone to develop a type 2 immune response that can contribute to or be responsible for fibrosis, while DSS colitis is marked by a type I response.^13^, ^35^, ^36^ Moreover, as ILC2 are scarce is the human intestine, both in normal and in IBD, contribution of these cells to fibrosis is made even more questionable.^35^

Interestingly, slower recovery of weight after repeated inflammatory insults by DSS exposure was observed in RAG^−/−^γc^−/−^ mice, suggesting a protective role for ILC. Protective effects of ILC via IL‐22 production have been shown in the recovery phase of acute intestinal injury.^37^ As almost no IL‐22 was produced upon ILC depletion in our experiments, this might contribute to the delayed recovery in ILC deficient mice, and further points to a protective effect of ILC probably via IL‐22 production and eventually other cytokines. However, as recovery was not impaired in ILC‐depleted mice, this effect is arguably due to impaired cytokine signaling in RAG^−/−^γc^−/−^ mice.

We also evaluated expansion of neutrophils, eosinophils, and monocytes in chronic DSS colitis in the different strains. All three cell types were indeed expanded in each of the strains and also after YTS treatment. These inflammatory cells might contribute and/or be essential for the induction of chronic intestinal remodeling in an ILC‐independent innate immune process. Upon intestinal injury, neutrophils are the first cells recruited into the intestinal lining as a first line of response.^25^ Neutrophils are short‐living cells but are still present up to 2 weeks after last DSS exposure in the chronic DSS model, suggesting their contribution not only to active inflammation but also to recovery and remodeling. In IBD patients, neutrophils accumulate around abscesses and contribute to cryptitis, but their presence does not correlate with strictures.^38^, ^39^ Importantly, studies in experimental pulmonary fibrosis have shown a neutrophil dependent IL‐17A pathway in the induction of lung fibrosis.^40^ However, IL‐17A neutralization in our model did not result in attenuation of intestinal fibrosis.^21^

The observed increase of eosinophils in RAG‐1^−/−^ but not in RAG^−/−^γc^−/−^ mice after three cycles of DSS can be explained by the absence of the common γc in the latter strain. Carlens et al^41^ could show a reduction of eosinophils specifically in the intestine in RAG^−/−^γc^−/−^ mice, together with a reduction in CCL11 (eotaxin‐1). Eosinophils can also be drivers of inflammatory damage and potentially contribute to tissue repair and remodeling in IBD.^42^, ^43^, ^44^, ^45^ Eosinophils, sources of IL‐4 and IL‐13, have been implicated in tissue remodeling in other diseases including eosinophilic oesophagitis, asthma, and hypereosinophilic syndrome.^44^, ^45^ More recently, eosinophils were identified as a crucial pathogenic source of intestinal fibrosis in radiation‐induced intestinal fibrosis.^46^ Furthermore, the role of an IL‐33‐eosinophil pathway was recently shown in pediatric CD strictures and these findings were validated in SAMP1/Yit mice.^47^

As administration of DSS leads to a disruption of the epithelial barrier and release of mature IL‐33, elevated levels of IL‐33 can be a potential driver of inflammation and remodeling in our model. However, IL‐33 plays a dual role as IL‐33^−/−^ mice showed an exacerbation of acute DSS colitis due to decreased levels of KC and MIP‐2 and an impaired neutrophil resolution of damage, while exogenous IL‐33 administration attenuated chronic DSS inflammation^48^, ^49^

In conclusion, our results show that DSS‐induced chronic murine colitis and fibrosis can develop independently of T cells, B cells, and ILC, pointing to an innate immune response mediated by neutrophils, macrophages, and/or eosinophils, as the drivers of inflammation and fibrosis. Our results do not exclude a contributing/modulatory role of ILC, especially of ILC2, but ILC2 are clearly not essential to the process of experimental intestinal fibrogenesis. On the other hand, ILC3 and their IL‐22 production might be protective against epithelial damage. We emphasize that these results were obtained in a particular murine model, which does not necessarily reflect the human situation. However, also in human IBD more data now support a primary role of myeloid cells.^6^, ^7^

## CONFLICT OF INTERESTS

Marc Ferrante received financial support for research from Amgen, Biogen, Pfizer, Takeda, and Janssen; lecture fees: Abbvie, Amgen, Biogen, Boehringer‐Ingelheim, Falk, Ferring, Janssen, Lamepro, MSD, Mylan, Pfizer, and Takeda; consultancy fees: Abbvie, Boehringer‐Ingelheim, Janssen, MSD, Pfizer, Sandoz, and Takeda. Séverine Vermeire reports financial support for research: MSD, AbbVie, Takeda, Pfizer, and J&J; Lecture fees: MSD, AbbVie, Takeda, Ferring, Centocor, Hospira, Pfizer, J&J, and Genentech/Roche; Consultancy: MSD, AbbVie, Takeda, Ferring, Centocor, Hospira, Pfizer, J&J, Genentech/Roche, Celgene, Mundipharma, Celltrion, SecondGenome, Prometheus, Shire, Prodigest, Gilead, and Galapagos. Gert Van Assche reports financial support for research from Abbott and Ferring Pharmaceuticals; lecture fees from Janssen, MSD, and Abbott; consultancy fees from PDL BioPharma, UCB Pharma, Sanofi‐Aventis, Abbott, Abbvie, Ferring, Novartis, Biogen Idec, Janssen Biologics, NovoNordisk, Zealand Pharma A/S, Millennium/Takeda, Shire, Novartis, and Bristol Mayer Squibb. Christine Breynaert reports consultancy fees from Ablynx.

## AUTHOR CONTRIBUTIONS

All authors made substantial contributions to the submitted work. BC contributed to the study concept and design, acquisition of data, analysis and interpretation of data, drafting of the manuscript, and statistical analysis. JC: acquisition data and interpretation of data. GDH: acquisition data, analysis and interpretation of data, and critical revision of the manuscript for important intellectual content. LB: material support. GvA: study concept and design, interpretation of data, and critical revision of the manuscript for important intellectual content. MF and SV: interpretation of data, and critical revision of the manuscript for important intellectual content. JLC: study concept and design, interpretation of data, and critical revision of the manuscript for important intellectual content. CB: study concept and design, analysis and interpretation of data, material support, drafting of the manuscript, and study supervision. All authors agreed with the final version of the manuscript. CB guarantor of the manuscript.

## Supporting information

Supporting information

## Data Availability

The data that support the findings of this study are available from the corresponding author upon reasonable request.
