# Supplementary material for: Fibrogenesis in chronic murine colitis is independent of innate lymphoid cells
Source: Immun Inflamm Dis. 2020 Jun 21;8(3):393–407. doi: 10.1002/iid3.321 (PMC7416052; doi:10.1002/iid3.321)
Supplement: Supplementary file 1 — Supporting information [file IID3-8-393-s001.docx]

**Supplementary data**

|  | **Target** | **Clone** | **Fluorophore** | **Isotype** | **Company** | **Productnumber** |
| --- | --- | --- | --- | --- | --- | --- |
| **ILC** | ***Lineage markers*** | | | | | |
|  | CD19 | 1D3 | FITC | Rat IgG k | BD | 557398 |
|  | CD3e | 145-2C11 |  | Hamster IgG k | BD | 553061 |
|  | CD45RB | 16A |  | Rat IgG k | ThermoFisher | 11-0455-82 |
|  | CD5 | 54-7-3 |  | Rat IgG k | BD | 553020 |
|  | CD94 | 18D3 |  | Rat IgG k | BD | 105506 |
|  | Gr-1 | RB6-8C5 |  | Rat IgG k | BD | 553126 |
|  | CD11b | M1/70 |  | Rat IgG k | BD | 557396 |
|  | TCR gd | GL3 |  | Rat IgG k | BD | 553177 |
|  | Ter-119 | TER-119 |  | Rat IgG k | BD | 557915 |
|  | ***ILC markers*** | | | | | |
|  | CD127 | 5B/199 | PE | Rat IgG k | BD | 552543 |
|  | CD45 | 30-F11 | BV510 | Rat IgG k | BD | 563891 |
|  | CD90.2 | 53-2;1 | PE-Cy7 | Rat IgG k | BD | 561541 |
|  | ***ILC Subset markers*** | | | | | |
|  | RORγt | AFKJS-9 | APC | Rat IgG k | ThermoFisher | 17-6988-82 |
|  | KLRG-1 | 2F1 | PerCP Cy5.5 | Rat IgG k | BD | 563595 |
|  | NK1.1 | PK136 | BV421 | Mouse IgG k | BD | 562921 |
| **Myeloid cells** | CD45 | 30-F11 | BV510 | Rat IgG k | BD | 563891 |
|  | CD11b | M1/70 | PE-Cy5 | Mouse IgG k | Biolegend | 101216 |
|  | Ly6G | 1A8 | BV711 | Mouse IgG k | Biolegend | 127643 |
|  | Ly6c | HK1.4 | PE | Mouse IgG k | Biolegend | 128008 |
|  | MHCII | 2G9 | FITC | Mouse IgG k | Biolegend | 553623 |
|  | Siglec F | E50-2440 | BV421 | Mouse IgG k | Biolegend | 562681 |
|  | CD64 | X54-5/7.1 | AF647 | Mouse IgG k | Biolegend | 139322 |
|  | CD117 | 2BA | AF700 | Rat IgG k | Biolegend | 105846 |
|  | FcεR | MAR-1 | PerCp Cy5.5 | Hamster IgG k | Biolegend | 134320 |
| **Intra-cell.** | IL-13 | eBio13A | PE | Rat IgG k | ThermoFisher | 46-7133-82 |

**Supplementary Table 1. Overview of antibodies used for detection of ILC and myeloid cells**

Innate lymphoid cell (ILC), cluster of differentiation (CD), Killer cell lectin-like receptor subfamily G member (KLRG), retinoic acid receptor-related orphan receptor gamma (RORyt), Interleukin (IL), T cell receptor (TCR), Immunoglobulin (Ig), Alexa fluor (AF), Allophycocyanin (APC), Brilliant Violet (BV), Fluorescein isothiocyanate (FITC), Fixable viability dye (FVD), Phycoerythrin (PE), Peridinin Chlorophyll Protein Complex (PerCP).

**
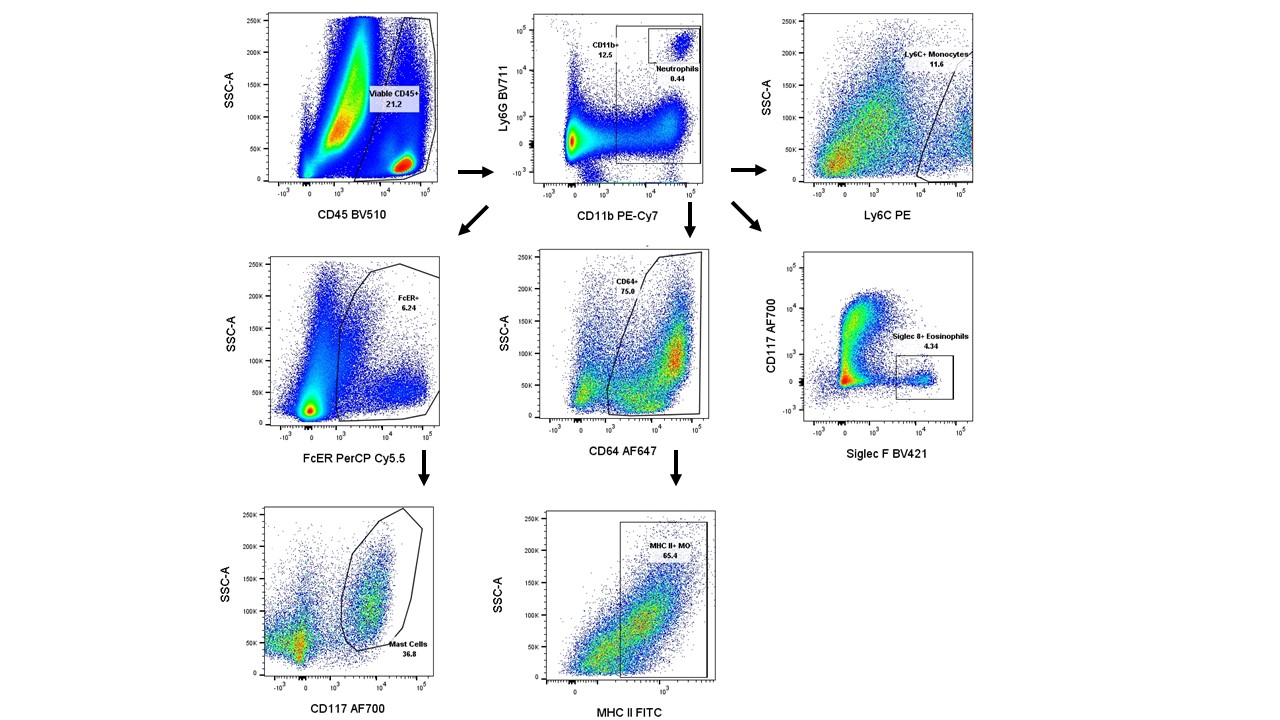
**

**Supplementary Figure 1: Gating strategy for myeloid cells**

Lamina propria cells were stained to define neutrophils (CD45^+^ CD11b^+^ Ly6G^+^), eosinophils (CD45^+^CD11b^+^ Siglec-8^+^), mast cells (CD45^+^CD11b^-^ CD117^+^ Fcεr^+^), macrophages (CD45^+^ CD11b^+^ CD64^+^MHCII^+^) and monocytes (CD45^+^CD11b^+^Ly6C^+^). Gating was based on FMO controls. As an, data of the lamina propria cells of a chronic DSS wild type mouse is shown. Dextran sulfate sodium (DSS), cluster of differentiation (CD), Major histocompatibility complex (MHC).

**
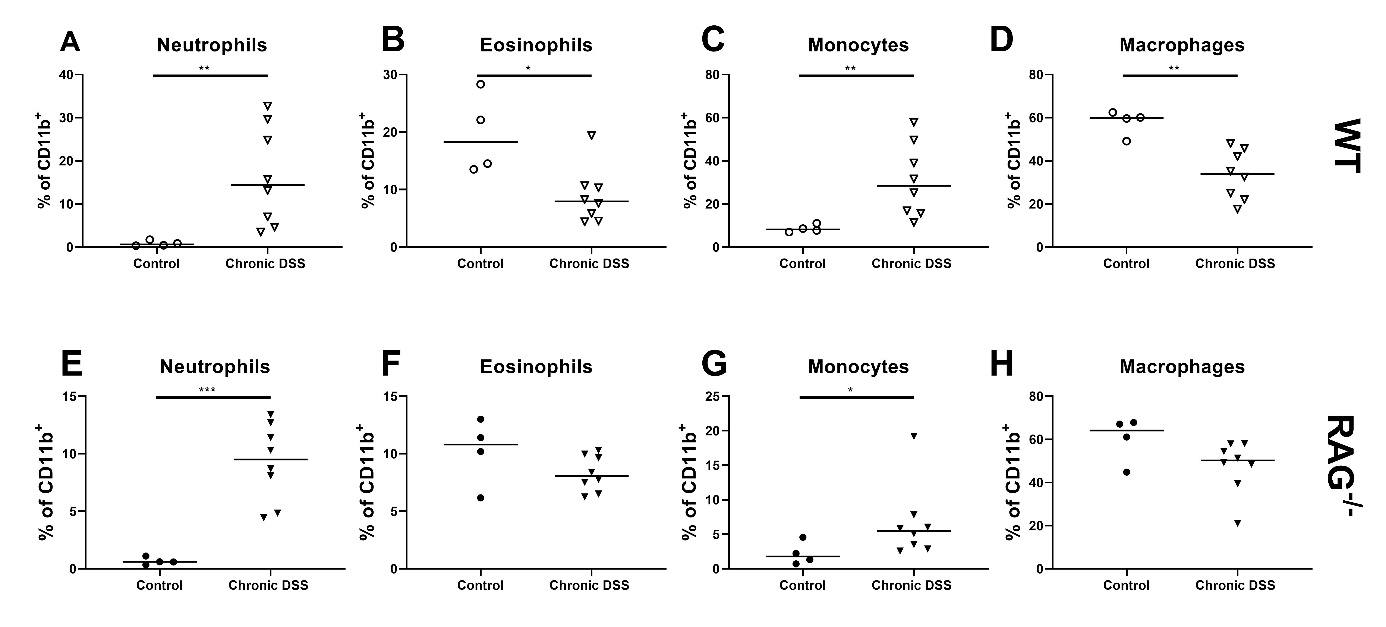
**

**Supplementary Figure 2: Effect of chronic DSS colitis on myeloid leukocyte populations in the distal colon of WT and RAG-1^-/-^ mice**

WT and RAG^-/-^ were exposed to three cycles of DSS (n=8) as compared to control mice on the same background without DSS administration (n=8). Different types of myeloid immune cells were identified by staining and flow cytometry on the distal lamina propria cells suspension from the distal colon. Relative contribution of (A) neutrophils, (B) eosinophils, (C) monocytes and (D) macrophages in in WT mice. Relative contribution of (E) neutrophils, (F) eosinophils, (G) monocytes and (H) macrophages in RAG^-/-^ mice. Results are expressed as % of the myeloid CD11b^+^ population. Mann-Whitney U testing within each background is shown; * p<0.05, ** p≤0.01, *** p≤0.001. Data are shown as individual values with median. Dextran sulfate sodium (DSS), Recombination activating gene (RAG).

**
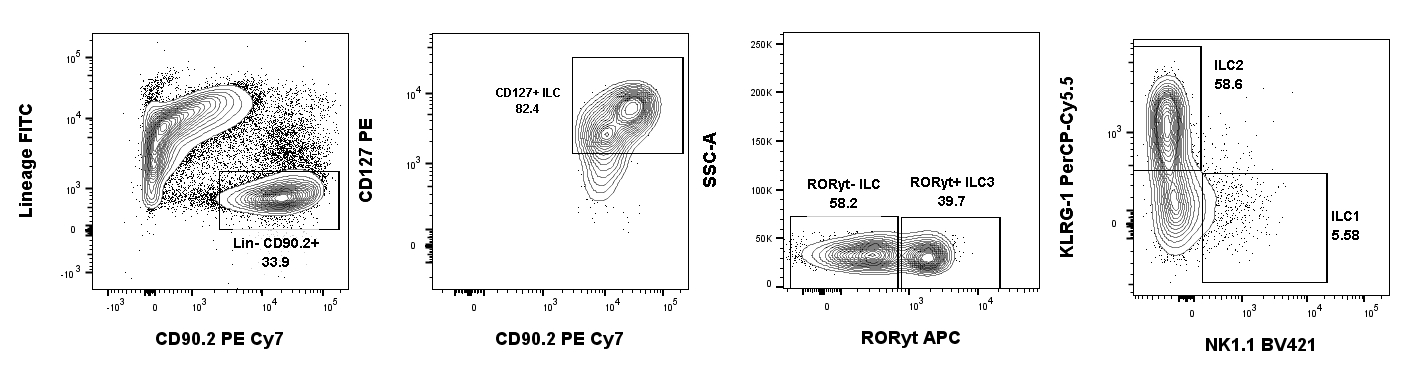
**

**Supplementary Figure 3: Gating strategy for innate lymphoid cells and their subsets**

Lamina propria cells were isolated and stained to study intestinal ILC and ILC subsets by flow cytometry. Within CD45^+^ living singlets, ILC were defined as lineage^-^ CD90.2^+^ CD127^+^ cells. Within the ILC, ILC3 were defined as RORyt^+^ by intracellular staining, ILC2 were defined as KLRG-1^+^RORyt^-^/dim and ILC1 were defined as KLRG-1^-^RORyt^-^NK1.1^+^ cells. Gating was based on FMO controls. As an example, data of the lamina propria cells of a chronic DSS colitis RAG^-/-^ mouse is shown. Calculated subset proportions for this example are 3.28 % ILC1, 34.11 ILC2 and 39.70% ILC3. Dextran sulfate sodium (DSS), Recombination activating gene (RAG), innate lymphoid cell (ILC), cluster of differentiation (CD), Killer cell lectin-like receptor subfamily G member (KLRG), retinoic acid receptor-related orphan receptor gamma (RORyt), Fluorescene minus one (FMO).


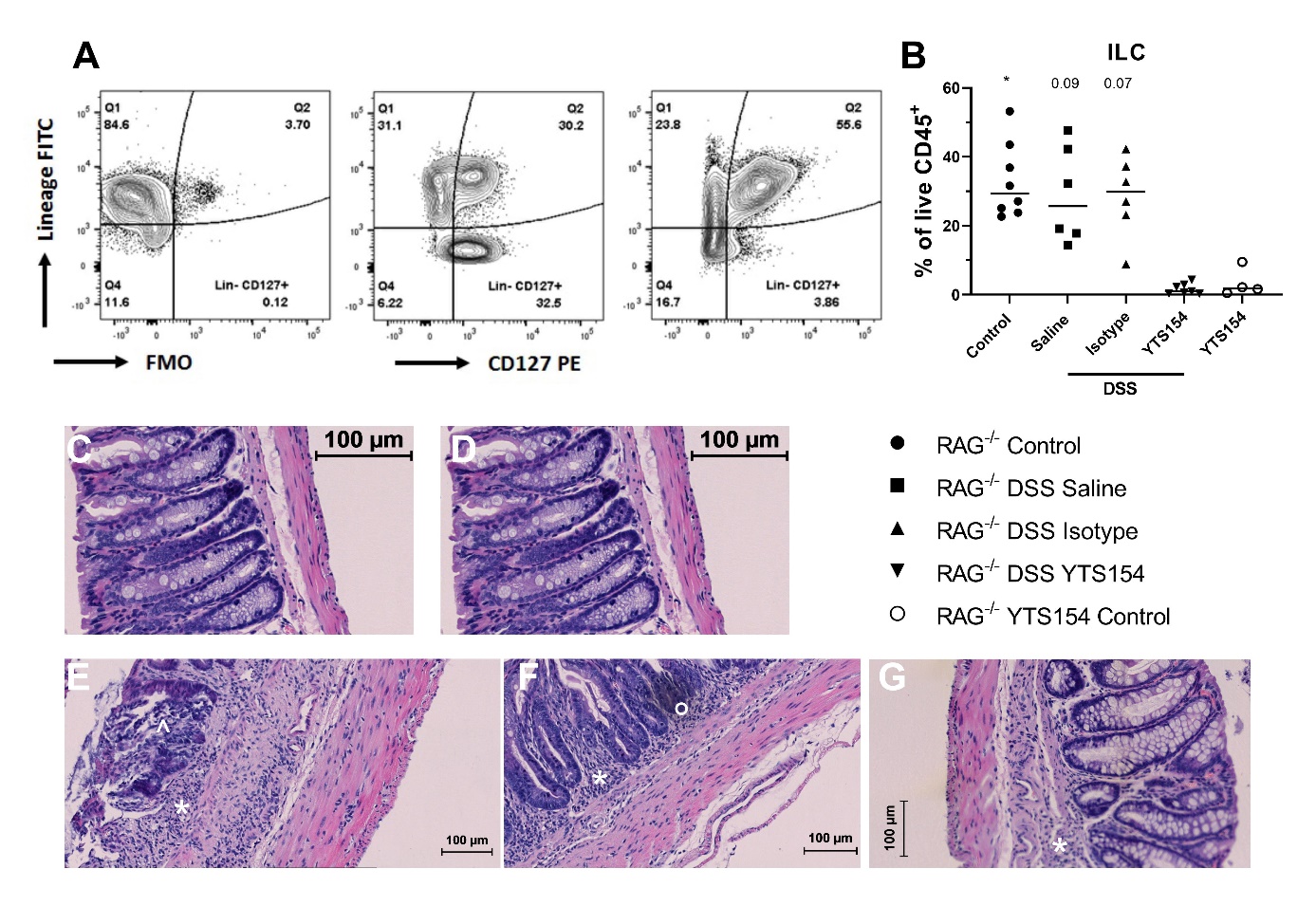


**Supplementary Figure 4: Anti-CD90 depletes ILC but does not result in an altered intestinal phenotype**

RAG^-/-^ were injected with saline (n=7), isotype (n=6) or YTS antibody (n=8) and exposed to three cycles of DSS and compared to control mice without DSS exposure (n=8) and YTS injected controls (n=4). ILC were identified as lineage^-^CD127^+^ cells within living CD45^+^ cells. (A) The gate for CD127+ cells was based on FMO (without the anti-CD127 mAb) on LP cells from a DSS exposed RAG^-/-^ mouse (left). The second and third dot pot are representative examples for identification of CD127^+^ ILC in LP cells of saline (middle) and YTS154 (right) injected DSS exposed RAG^-/-^  mice. (B) Proportion of ILC as % of total CD45^+^ leukocytes in the five groups of mice as indicated. (C-G) Representative hematoxylin and eosin stainings of control mice without DSS exposure (C), YTS injected control mice (D) and saline (E), isotype (F) and YTS (G) injected chronic DSS colitis mice. *Cell infiltration, ^loss of crypt architecture, °cryptitis. Cluster of differentiation (CD), Dextran sulfate sodium (DSS), Fluorescence Minus One (FMO), innate lymphoid cell (ILC), Lamina Propria (LP), Recombination activating gene (RAG).
